# Rapid genetic typing of diarrheagenic *Escherichia coli* using a two-tube modified molecular beacon based multiplex real-time PCR assay and its clinical application

**DOI:** 10.1186/s12941-014-0030-8

**Published:** 2014-07-15

**Authors:** Qingliang Chen, Xiaolu Shi, Yinghui Li, Yixiang Jiang, Yiman Lin, Yaqun Qiu, Qingge Li, Qinghua Hu

**Affiliations:** 1 Engineering Research Center of Molecular Diagnostics, Ministry of Education, Department of Biomedical Sciences, School of Life Sciences, Xiamen University, Xiamen 361005, Fujian Province, China; 2 Shenzhen Major Infectious Disease Control Key Laboratory, Shenzhen Centre for Disease Control and Prevention, Shenzhen 518055, Guangdong Province, China; 3 School of Life Sciences, Shenzhen University, Shenzhen 518000, Guangdong, China; 4 Shenzhen Research Institute of Xiamen University, Shenzhen 518057, Guangdong, China

**Keywords:** Diarrheagenic Escherichia coli, Two-tube multiplex real-time PCR, Modified molecular beacon, Identification, Application

## Abstract

**Background:**

Diarrheagenic *Escherichia coli* (DEC), including *Enterotoxigenic E.coli* (ETEC), *Enteroaggregative E.coli* (EAEC), *Enteropathogenic E.coli* (EPEC), *Enterohemolysin E.coli* (EHEC) and *Enteroinvasive E.coli* (EIEC) causes diarrhea or hemolytic uremic syndromes among infants and travelers around the world. A rapid, reliable and repeatable method is urgent for identifying DEC so as to provide the reference for responding to diarrheal disease outbreak and the treatment of the diarrheal patients associated with DEC.

**Methods:**

In this study, specific primers and modified molecular beacon probes of nine specific virulence genes, whose 5′end were added with homo tail sequence, were designed; and a two-tube modified molecular beacon based multiplex real–time PCR (rtPCR) assay for the identification of five *Escherichia coli* pathotypes, including ETEC, EAEC, EPEC, EHEC and EIEC was developed and optimized. Totally 102 bacterial strains, including 52 reference bacterial strains and 50 clinical strains were detected to confirm whether the target genes selected were specific. Then detection limits of the assay were tested. Lastly, the assay was applied to the detection of 11860 clinical samples to evaluate the specificity and sensitivity of the developed assay compared with the conventional PCR.

**Results:**

The target genes were 100% specific as assessed on 102 bacterial strains since no cross-reactions were observed. The detection limits ranged from 88 CFU/mL (EHEC) to 880 CFU/mL (EPEC). Compared with the conventional PCR, the specificity and sensitivity of the multiplex rtPCR was 100% and over 99%, respectively. The coefficient of variation (CV) for each target gene ranged from 0.45% to 1.53%. 171 positive clinical samples were mostly identified as ETEC (n = 111, 64.9%) and EPEC (n = 38, 22.2%), which were the dominating pathotypes of DEC strains.

**Conclusion:**

The developed multiplex rtPCR assay for the identification of DEC was high sensitive and specific and could be applied to the rapid identification of DEC in clinical and public health laboratories*.*

## Background

Diarrheal disease has been considered as one of the most important public health problem, as well as the significant cause leading to children’s death [[1]] and brings high disease burden to society [[2]]. Diarrheagenic *Escherichia coli* (DEC) is the main pathogen associated with diarrheal disease.

Based on the epidemiological, clinical features, specific virulence determinants and other molecular characteristics, DEC has been classified into five main pathotypes as follows: *Enterotoxigenic E.coli* (ETEC), *Enteroaggregative E.coli* (EAEC), *Enteropathogenic E.coli* (EPEC), *Enterohemolysin E.coli* (EHEC) and *Enteroinvasive E.coli *(EIEC) [[2]]. Each pathotype possesses specific virulence genes associated with the disease symptoms. For example, shiga-like toxin I (*stx1*), shiga-like toxin II (*stx2*), *eaeA* and *escV* genes are virulence genes for EHEC. *EaeA* encodes intimin that involved in attachment of bacteria to enterocytes, and *escV* is the main component of ring structure on inner membrane. EPEC also contains *eaeA* and *escV*, but without shiga-like toxin [[3]]. For ETEC, it carries the plasmid encoded heat-stable (ST) and/or heat-labile (LT) toxins. ST can be divided into two main groups, *stp* (also referred as *STIa*) and *sth* (also referred as *STIb*) [[4]]. Gene *ipaH*, which is related to invasion [[5]] expressed in EIEC and *shigella spp.*. The defining feature of EAEC is the distinctive aggregative-adherence pattern, which is mediated by *aggR* gene [[6]].

DEC will mostly result in persistent watery diarrhea, abdominal cramping and diarrhea. EHEC, such as O157:H7 will cause hemolytic uremic syndrome (HUS) and is life-threatening. DEC is also associated with foodborne disease outbreak or infantile diarrheal disease outbreak. So identification of different pathotypes of DEC will provide the critical information for determining appropriate therapies for patients with suspected *E.coli* infections and controlling of the outbreak. A variety of assays have been published for identification of DEC. Serotyping and biochemistry has been widely applied in the diagnosis of gastrointestinal pathogens, but they cannot be used for conclusive identification of DEC groups [[7]]. Molecular assays [[8]-[18]], like DNA hybridization [[8]], loop-mediated isothermal amplification (LAMP) [[9]], multiplex PCR [[10],[11],[15],[17],[18]], and real-time PCR (rtPCR)-SYBR green melting curve [[12]-[14]] have been described for such identification. Among these techniques, multiplex PCR is one of the most useful diagnostic methods, however, this method needs post gel electrophoresis, which is labor-consuming and can easily result in carry-over contamination. Thus it is not suitable for analyzing large numbers of clinical samples in clinical laboratory. Guion et al. [[14]] creatively used rtPCR SYBR green melting-curve to detect eight virulence gene of DEC, and the results could be obtained from the peak of melting temperature(T_m_). However, SYBR green melting-curve is not a stable method, since it’s prudent to standardize the assay using control strains in each laboratory.

We have developed a multiplex rtPCR assay based multicolor combinational probe coding (MCPC) for the identification of eight foodborne pathogens [[19]] and applied to the rapid diagnosis of foodborne disease outbreak successfully. In order to quickly screen DEC from large number of stool samples in laboratory-based surveillance network of diarrheal disease and respond to diarrheal disease outbreak associated with DEC, in this paper, based on the principle of MCPC, we attempted to design modified molecular beacons and primers with homo tail sequences and also add internal amplification control to establish the multiplex real-time PCR assay for the identification of DEC. Considering the different modes of the real-time PCR instruments used in the different hospitals, we established a two-tube multiplex rtPCR assay for detecting nine genes simultaneously with the same thermocycling condition and DEC can be typed within 90 min.

## Results

### Detection limits and reproducibility of multiplex rtPCR

**Figure 1:**
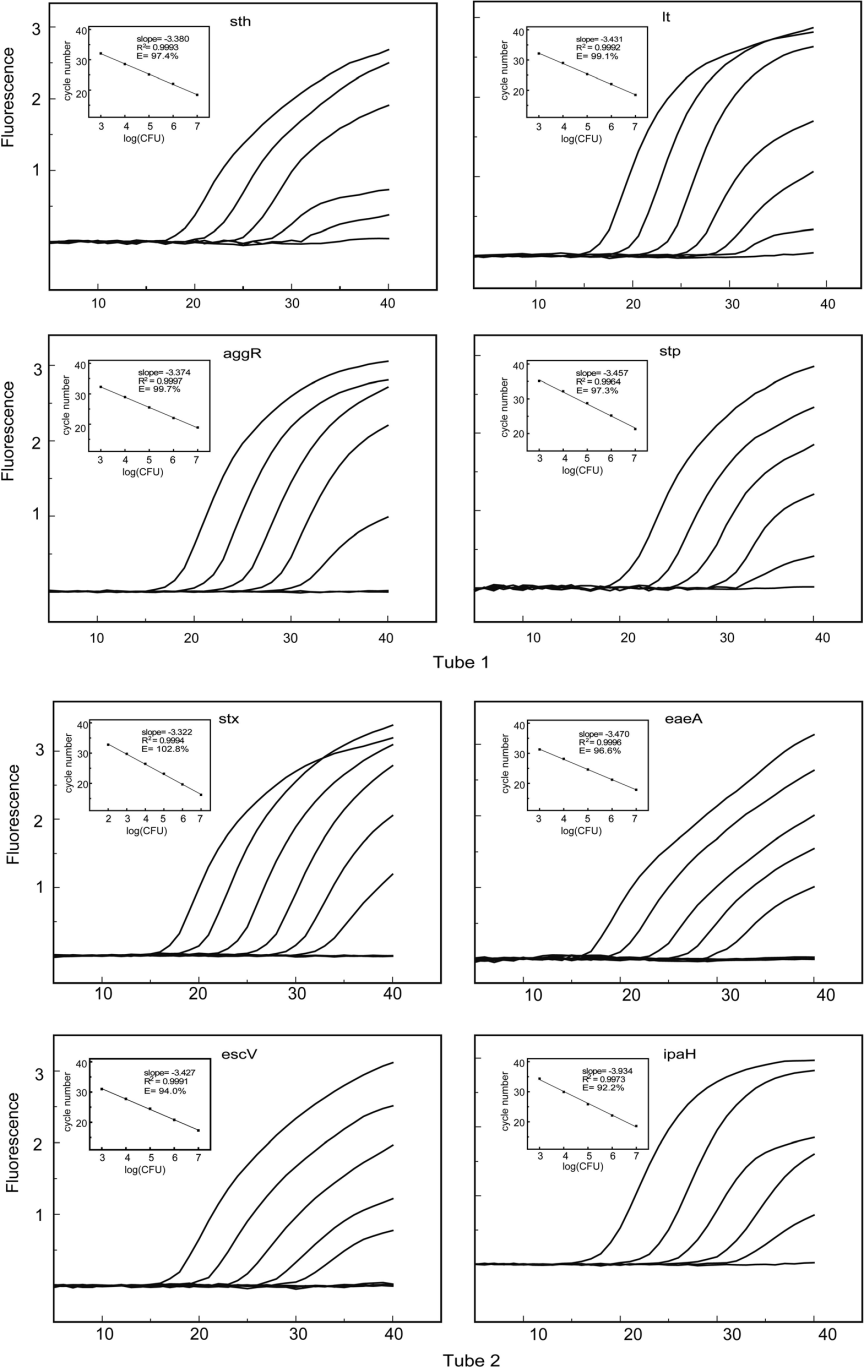
**Detection limits of five main pathotypes of diarrheagenic** ***Escherichia coli*.** Ten-fold serial dilutions of bacterial cultures of five diarrheagenic *Escherichia coli* pathotypes were prepared to assess the detection limits.

The detection limits of this method for bacterial pure culture ranged from 88 CFU/mL (EHEC) to 880 CFU/mL (EPEC). Amplification efficiencies for bacterial culture ranged from 92.2% (EIEC) to 102.8% (EHEC) (Figure 1 and Table 1).

**Table 1:** Detection limits and amplification efficiency of multiplex rtPCR assay

**Tube**	**Pathotypes**	**Virulence gene**	**Equation**	**R**^**2**^	**Efficiency**	**Liner range (CFU/mL)**	**Detection limits (CFU/mL)**
Tube 1	ETEC	*stp*	y = −3.345 × +45.797	0.9964	97.3%	3.0 × 10^2^ ~ 3.0 × 10^6^	3.0 × 10^2^
		*sth*	y = −3.386 × +42.136	0.9993	97.4%	3.0 × 10^2^ ~ 3.0 × 10^6^	3.0 × 10^2^
		*lt*	y = −3.343 × +42.537	0.9992	99.1%	3.0 × 10^2^ ~ 3.0 × 10^7^	3.0 × 10^2^
	EAEC	*aggR*	y = −3.371 × +42.398	0.9997	99.7%	2.0 × 10^2^ ~ 2.0 × 10^6^	2.0 × 10^2^
Tube 2	EHEC	*stx1/2*	y = −3.257 × +39.610	0.9994	102.8%	8.8 × 10^1^ ~ 8.8 × 10^6^	8.8 × 10^1^
	EPEC	*eaeA*	y = −3.405 × +41.464	0.9996	96.6%	8.8 × 10^2^ ~ 8.8 × 10^6^	8.8 × 10^2^
		*escV*	y = −3.475 × +41.401	0.9991	94.0%	8.8 × 10^2^ ~ 8.8 × 10^6^	8.8 × 10^2^
	EIEC	*ipaH*	y = −3.523 × +45.832	0.9973	92.2%	6.8 × 10^2^ ~ 6.8 × 10^6^	6.8 × 10^2^

The C_T_ values from three replicate experiments for each ten-fold dilution (10^6^ cfu/ml -10^3^ cfu/ml) differed within one cycle and the coefficient of variation (CV) for each target gene ranged from 0.45% to 1.53%, which indicated that the assay was reproducible (Table 2).

**Table 2:** The reproducibility of the two-tube multiplex real-time PCR assay

**genes**	**10**^**6**^ **cfu/ml**		**10**^**5**^ **cfu/ml**		**10**^**4**^ **cfu/ml**		**10**^**3**^ **cfu/ml**	
	**Mean Ct & SD**	**CV (%)**	**Mean Ct & SD**	**CV (%)**	**Mean Ct & SD**	**CV (%)**	**Mean Ct & SD**	**CV (%)**
eaeA	21.22 ± 0.18	0.85	24.58 ± 0.22	0.90	28.14 ± 0.31	1.10	31.26 ± 0.26	0.83
escV	20.8 ± 0.13	0.63	24.49 ± 0.11	0.45	27.73 ± 0.25	0.90	30.99 ± 0.32	1.03
ipaH	22.11 ± 0.18	0.81	25.85 ± 0.31	1.12	29.93 ± 0.22	0.74	34.34 ± 0.458	1.31
Stx1/2	19.64 ± 0.18	0.92	23.16 ± 0.09	0.39	26.44 ± 0.21	0.79	29.74 ± 0.2	0.67
aggR	22.02 ± 0.17	0.77	25.52 ± 0.31	1.21	28.95 ± 0.39	1.35	32.3 ± 0.28	0.87
lt	22.06 ± 0.14	0.63	25.29 ± 0.31	1.23	28.99 ± 0.22	0.76	32.13 ± 0.49	1.53
sth	22.04 ± 0.0.13	0.58	25.11 ± 0.31	1.23	28.54 ± 0.4	1.40	32.07 ± 0..25	0.78
stp	25.2 ± 0.09	0.36	28.76 ± 0.29	1.01	32.19 ± 0.23	0.71	35.1 ± 0.21	0.60

### Specificity of target genes for multiplex rtPCR

All 102 bacterial strains were correctly identified and no cross-reaction or false positives were observed, demonstrating that the selected target genes were specific for their respective pathotypes (Additional file 1: Table S1).

### Application of multiplex rtPCR for the identification of suspected DEC samples

Of the 11860 stool samples, 5100 samples were putatively E.coli positive on MacConkey agar and biochemical test. Among 5100 suspected DEC samples, 171 were DEC positive, including 38 ETEC, 111 EPEC, four EHEC, 10 EAEC and eight EIEC identified by multiplex rtPCR assay, whereas 150 were DEC positive including 29 ETEC, 99 EPEC, four EHEC, 10 EAEC and eight EIEC were identified by the conventional PCR.

When compared with the conventional PCR, the sensitivity of the multiplex rtPCR assay was 100% (95% CIs 39.76-100%) for all five pathotypes detected, and the specificity for ETEC, EPEC, EHEC, EAEC, and EIEC was 99.92%, 99.90%, 100%, 100% and 100%, respectively (95% CIs 99.82-100%). Kappa value for ETEC, EPEC, EHEC, EAEC, and EIEC was 0.87, 0.94, 1.00, 1.00 and 1.00, respectively (Table 3).

**Table 3:** Sensitivity and specificity tested using multiplex real-time PCR and conventional PCR in 11860 clinical samples

**DEC group**	**Multiplex real-time PCR**	**Standard PCR**		**Total**	**Sensitivity**	**95% CI**	**Specificity**	**95% CI**	***Kappa***
		**+**^**1**^	**−**^**2**^						
ETEC	+	29 (a)	9 (b)	38	100%	88.06 ~ 100.00	99.92%	99.86 ~ 99.97	0.87
	-	0 (c)	11822 (d)	11822					
	Total	29	11831	11860					
EPEC	+	99	12	111	100%	96.34 ~ 100.00	99.90%	99.82 ~ 99.95	0.94
	-	0	11749	11749					
	Total	99	11761	11860					
EHEC	+	4	0	4	100%	39.76 ~ 100.00	100%	99.97 ~ 100.00	1.00
	-	0	11856	11856					
	Total	4	11856	11860					
EAEC	+	10	0	10	100%	69.15 ~ 100.00	100%	99.97 ~ 100.00	1.00
	-	0	11850	11850					
	Total	10	11850	11860					
EIEC Positive	+	8	0	8	100%	63.06 ~ 100.00	100%	99.97 ~ 100.00	1.00
	-	0	11852	11852					
	Total 171	8 150	11852	11860					

+^1^: Positive, −^2^: Negative.

### Distribution of DEC pathotypes among 171 DEC positive clinical samples

**Figure 2:**
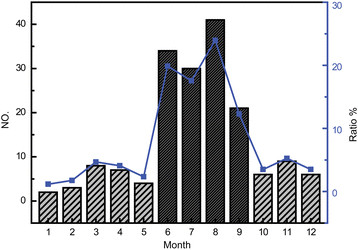
**Monthly distribution (in bar) and ratio (point) of diarrheagenic** ***Escherichia coli*** **clinical isolates in 2011 to 2012.**

Among 171 DEC positive samples, the positive rate of ETEC and EPEC were 64.9% (111/171) and 22.2% (38/171), respectively. And the positive rate of EAEC, EIEC, EHEC were 5.8% (10/171), 4.7% (8/171) and 2.3% (4/171), respectively. Besides, the data showed that adults were the main infected group, which accounted for 82.5% (141/171). The infection rate of children (age ≤ 6 year) was only 12.9% (22/171). Obvious monthly peaks of DEC infections were found during the warmer months from June to September; approximately 73.7% (126/171) of diarrheal patients could be attributed to DEC infections during this period (Figure 2).

## Discussion

In present study, two-tube multiplex rtPCR was successfully established for identifying five pathotypes of DEC using homo tail primers and modified molecular beacons simultaneously. The detection limits ranged from 88 CFU/mL to 880 CFU/mL. Compared with the conventional PCR assay, detection data of 11860 clinical samples showed that two-tube multiplex rtPCR possessed 100% sensitivity and above 99% specificity. After optimizing cycling condition of the assay, five categorized DEC were detected at the same time, leading to a great reduction of cost and time. And the time required from DNA extraction to final result was less than 90 min. Therefore the assay was a high sensitive, specific and rapid method.

Traditionally, high throughput rtPCR was restricted by the wavelength of fluorescence channel and primer dimer formation, which may result in simultaneous detection of two or three genes in one reaction. The follows were used as means to overcome these limitations: First, part of the stem structure of probes was designed to participate in hybridization of template to increase the binding affinity and reduce the background of fluorescence signal greatly. Second, to reduce dimer formation, homo-tag assisted non-dimer (HAND) method [[20],[21]] was put into use to ensure the stability and sensitivity of multiplex rtPCR. Also, the additional tag sequence increased the T_m_ value of primer, making the optimization of the same cycling temperature for two-tube assay easier. The above methods are able to enlarge the signal-to-noise ratio, and enhance the efficiency of amplification. Comparing to gel electrophoresis and SYBR green melting curve, the use of fluorescence probes would avoid reopening the reaction tubes, thus greatly reduce the risk for carry-over contamination and false-positive and will also avoid errors from band discrimination when observed by naked eyes.

Comparing to the conventional PCR for DEC identification according to diagnostic criteria for infectious diarrhea (WS 271–2007, China) and other literature data, our assay covers more genes, such as *escV, stp* and *sth*. Since *eaeA* had a lot of single nucleotide polymorphisms, *escV* was also included for the identification of EPEC. The twelve extra positive samples were identified when *escV* was added. In addition, only using *st* gene without differentiation of *stp* and *sth* will lead to nine ETEC missing. Statistical analysis revealed that there was no difference among positive result of EAEC, EHEC and EIEC using multiplex rtPCR and the conventional PCR. All these suggested that more genes were preferred to improve the sensitivity of DEC identification method.

In order to indicate false-negative of multiplex rtPCR, an internal amplification control (IAC) was added to the multiplex rtPCR assay. We used the sequence of the human adenovirus (accession No. AY601634) as an internal amplification control to guarantee that proper reagents, such as magnesium, *Taq* polymerase and 1 × PCR buffer were suitable for PCR amplification.

Considering that clinical DEC strains should be isolated for the further antibiotics susceptibility test, we adapted the multiplex rtPCR to the suspected colonies for the identification of DEC, not directly to the fresh stool samples. Based on the research data of Barletta et al. [[12]], we used five-colony pool analysis from one clinical sample for typing DEC.

Epidemiological information of out-patients was collected, including patients’age and symptoms (data not shown). Among 171 positive cases, adults were the main infected population instead of children. This may be due to the population structure of Shenzhen, in which young workers constitute a big percentage of population. For DEC cases, ETEC was the predominant type, accounting for 64.9% of all cases. This was corresponded to a new systematic review [[22]]. During summer, diarrhea cases caused by DEC increased rapidly (Figure 2), suggesting a high risk of infection in summer and an essential measure to take to promote health care.

In conclusion, a rapid and effective two-tube multiplex rtPCR for the identification of all DEC pathotypes was established. This method was time (90 min) and resources saving (including biochemical reagents, disposable medical supplies) and could be used in clinical and public health laboratories.

## Materials and methods

### Reference bacterial strains and clinical strains

A total of 111 bacterial strains, comprising 24 species, including 61 reference bacterial strains from American Type Culture Collection (ATCC) or National Center for Medical Culture Collections of China (CMCC) and 50 clinical isolates, were collected as specific or nonspecific targets (Additional file 1: Table S1). Nine specific reference strains were selected for establishing multiplex rtPCR (four types of *shigella spp*.: CMCC 51265, CMCC 51376, ATCC 12022, CMCC 51081; ETEC: ATCC 35401; EAEC: ATCC 33780; EIEC: ATCC 43893; EHEC: ATCC 35150; and EPEC: ATCC 43887). All strains were confirmed by diagnostic criteria for infectious diarrhea published by Chinese Centre for Disease Control and Prevention (WS 271–2007) using the conventional PCR (Additional file 1: Table S2).

### Bacterial culture and DNA extraction

All positive reference bacterial strains were cultured on appropriate agar plates to isolate bacterial colonies. After overnight incubation at 37°C, single colonies of each culture were carefully picked and added into 200 μl sterile purified water. After shaking thoroughly and heating for five minute, slurry was centrifuged at 12000 rpm for one minute, and 5 μl supernatant was collected for PCR reaction.

Clinical stool samples were streaked on MacConkey agar plates. After overnight incubation at 37°C, a pool of five suspicious colonies was picked for DNA extraction as follows. A pool of five suspicious colonies was added to 200 μl sterile purified water and shaked thoroughly following heating lyses for five minute. After centrifuging at 12000 rpm for one minute, 5 μl supernatant was assimilated for PCR reaction.

### Primers and probes

Specific virulence genes were chosen to detect specific pathogens: *ipaH* for EIEC, *stp/sth/lt* for ETEC, *eaeA*/*escV* for EPEC, *stx1/stx2* for EHEC, *aggR* for EAEC. The sequence of the human adenovirus (accession No.AY601634) was selected as an internal amplification control(IAC) for the multiplex rtPCR assay [[23]]. All the sequences of genes published in the Genbank were downloaded and aligned by Clustal X 2.0. Conserved regions were picked up for designing primers and probes after merging into GenBank. Based on the articles published online [[24]], molecular beacon probes were designed with the help of DNA folding form website (http://mfold.rit.albany.edu/?q=mfold/DNA-Folding-Form). All the 5′end of primers were added with a homo tail sequence to alleviate the forming of primer dimer. Molecular beacons were modified and designed. All primers and probes were listed in Table 4.

**Table 4:** **The primers and probes used for multiplex real-time PCR assay in this study**^**a**^**F/R/P: forward and reverse primer,**^**b**^**concentration**

**Tube**	**Group**	**Target genes**	**Primers and Probes**^**a**^	**Sequence(5′-3′)**	**Conc**^**b**^**(μM)**
Tube 1	ETEC^1^	*stp*	*stp-*F	tag-AAAAGCGAGTGTACCTCGACA	0.18
			*stp-*R	tag-CAGTTGACTGACTAAAAGAGGGG	0.3
			*stp-*P	HEX-CGCGTCTCAAATATCCGTGAAACAACATGACGCG-Dabcyl	0.2
		*sth*	*sth-*F	tag-GTGGTCCTGAAAGCATGAATAG	0.12
			*sth-*R	tag-CAACAAAGCAACAGGTACATACG	0.12
			*sth-*P	FAM-CGCGGTGAATTGTGTTGTAATCCTGCTTGTACCGCG-Dabcyl	0.2
		*lt*	*lt-*F	tag-ACAGGAGGTTTCTGCGTTAG	0.18
			*lt-*R	tag-GGTGGGAAACCTGCTAATCT	0.18
			*lt-*P	ROX-CGCCGGTATTACAGAAATCTGAATATAGCTCCGGCG-Dabcyl	0.2
	EAEC^1^	*aggR*	*aggR-*F	tag-TGCAAAAGAAGAAATCAACAGT	0.18
			*aggR-*R	tag-CAGAATCGTCAGCATCAGCTAC	0.3
			*aggR-*P	CY5-CGGACAAAAGTAGATGCTTGCAGTTGTCCG-Dabcyl	0.2
Tube 2	EIEC^2^	*ipaH*	*ipaH-*F3	tag-GAAAACCCTCCTGGTCCATC	0.32
			*ipaH-*R3	tag-GTCTGGAAGGCCAGGTAGACTT	0.32
			*ipaH-*P	FAM-CCCGGCTGGAGGACATTGCCCGGG-Dabcyl	0.2
	EPEC&	*escV*	*escV-*F	tag-GGCTCTCTTCTTCTTTATGGCTG	0.4
	EHEC^2^		*escV-*R	tag-GGGAAAGAAGTTAGTTCAAGAGGAT	0.4
			*escV-*P	HEX-CCCGCGCAACAGTTGTGGTGGATATCATTATCGCGGG-Dabcyl	0.2
		*eaeA*	*eaeA-*F	tag-GTAACCAGGCTTCGTCACA	0.32
			*eaeA-*R	tag-AAGGAAAAAACGCTGACCCG	0.32
			*eaeA-*P	CY5-CCCAGTGGTAATAACTTTGACGGTAGTTCACTGGG-Dabcyl	0.2
	EHEC^2^	*stx1*	*stx1-*F	tag-ASAGCGGTTACATTGTCTGGT	0.24
			*stx1-*R	tag-CTGCGTCAGTGAGGTTCCA	0.24
			*stx1-*P	ROX-CCGCGTACGGGGATGCAGATAAATCGCGG-Dabcyl	0.2
		*stx2*	*stx2-*F	tag-CATGACAACGGACAGCAGTTA	0.24
			*stx2-*R	tag-TCTGGTCATTGTATTACCACTGAA	0.24
			*stx2-*P	ROX-CCGCCACTCACTGGTTTCATCATATCTGGCGG-Dabcyl	0.2
Tube 1&2	IAC^1,2^		IAC-F	GGCGCGCCTAACACATCT	0.1
			IAC-R	TGGAAGCAATGCCAAATGTGTA	0.1
			ICA-P	CGGTGGTTACAACGGGAGAAGACAATGCCACCG	0.2
			tag	GCAAGCCCTCACGTAGCGAA	2.4

^1^: tube1, ^2^: tube2.

*IAC*: Internal amplification control.

### Performance of multiplex rtPCR

The homo tail was added to 5′ end of primer sequences so that proper annealing temperature will fall into broad range in each individual PCR reaction. Five classes of DEC were divided into two tubes. For tube-one, *stp/sth/lt* for ETEC, *aggR* for EAEC and IAC were included, while *ipaH* for EIEC, *eaeA*/*escV* for EPEC, *stx1/stx2* for EHEC and IAC were included in tube-two. Multiplex rtPCR reactions were performed on Bio-Rad CFX 96 Real-Time PCR System (Bio-Rad, Hercules, CA). Firstly, individual real-time PCR cycling condition and proper reagent concentration were optimized. Secondly, two-tube system reagents, including primers, probes, magnesium and Taq polymerase are mixed and optimized separately. The optimal assay was as follows: tube-one, 1 × PCR buffer, 3 mM MgCl_2_, 2 μl of deoxynucleoside triphosphate (2.5 mM), and 1 unit Taq polymerase, 0.12 μM to 0.3 μM specific primers and probes (Table 4) and 5 μl of DNA template; tube-two, 1 × PCR buffer, 2.5 mM MgCl_2_, 2 μl of deoxynucleoside triphosphate (2.5 mM), and 1 unit Taq polymerase, 0.2 μM to 0.4 μM specific primers and probes (Table 4) and 5 μl of DNA template. The amplification cycles consisted of a first stage of denature at 95°C for 3 min; a second stage of 5 cycles: 95°C for 5 s, 58°C for 15 s, 72°C for 15 s; a third stage of 40 cycles: 95°C for 5 s, 55°C for 15 s, 72°C for 15 s. Carboxy fluorescein (FAM), Hexachloro fluorescein (HEX), Carboxy-X-rhodamine (ROX), Quasar 705 and indodicarbocyanine5 (Cy5) fluorescence were collected and recorded at the end of annealing step during the third stage.

### Detection limits and reproducibility of multiplex rtPCR

Five ATCC reference strains (ETEC ATCC 35401, EAEC ATCC 33780, EIEC ATCC 43893, EHEC ATCC 35150 and EPEC ATCC 43887) were used for the analysis of detection limits. The target strains were cultured in Luria broth and the concentration of the bacteria was adjusted to 10^6^ or 10^7^ colony forming units (CFU)/mL using a turbidity meter, then 1 mL enrichment broth was used as the dilution, meanwhile an aliquot of each dilution was quantified via plate counting. The DNA template of serial 1:10 dilutions was extracted by heating lyses method mentioned above and was used for the detection limits analysis and four ten-fold serial dilutions (10^6^-10^3^ cfu/μL) were prepared for the analysis of the reproducibility in three replicate experiments.

### Specificity of multiplex rtPCR assay

To confirm whether the target genes selected were specific for each pathotype of DEC, 102 well-characterized strains were tested by multiplex rtPCR.

### Application of multiplex rtPCR for the identification of suspected clinical samples

The multiplex rtPCR was applied to detecting clinical samples to determine assay sensitivity and specificity with direct comparison with conventional PCR. All clinical samples were collected based on the inclusion criteria of World Health Organization (WHO) case definition for diarrhea. Diarrheal disease was described as the follows: having three or more loose or liquid stools per day, or as having more stools than normal person. The stool samples of totally 11860 out-patients were collected for DEC detection from 11 sentinel hospitals during 2011 to 2012 in Shenzhen, Guangdong province, China.

All specimens were streaked onto MacConkey agar and a minimum of five typical suspected E.coli colonies were picked and identified by biochemical test and the conventional PCR according to the diagnostic criteria for infectious diarrhea (WS 271–2007, China). Then DNA of a pool of five typical suspected colonies was extracted and performed for both two-tube multiplex rtPCR and conventional PCR simultaneously. For conventional PCR, the reaction volume and amplification condition were as the follows: 5 μl of DNA template was applied to PCR reaction at the final volume of 50 μl; the PCR reaction consisted of 10 mM Tris–HCl (pH 8.3), 50 mM KCl, 0.1% Triton X-100, 1.5 mM MgCl_2_, 2.5 U Taq, 0.2mMdNTP, and 0.125-0.5 μM primer pairs. The cycling condition of PCR contained 95°C for 10 min; and 35 cycles of 94°C for 1 min, 52°C for 1 min, 72°C for 1 min; then 72°C for 10 min. PCR products were analyzed with 1.5% agarose gel electrophoresis. The primer sequences were attached in Additional file 1: Table S2.

### Statistical analysis

The results of the multiplex rtPCR assay were compared with conventional PCR using a 2 × 2 table to estimate indices of sensitivity and specificity. Corresponding 95% confidence intervals (CIs) were calculated by using approximate normal distribution and exact interval methods and Kappa value was calculated.

## Competing interests

The authors declare that they have no competing interests.

## Authors' contributions

QC designed primers and modified molecular beacons of target genes for EIEC and EPEC and carried out multiplex real-time PCR assay. XS designed primers and modified molecular beacons of target genes for EAEC and EHEC. YL participated in traditional PCR assay. YJ designed primers and modified molecular beacons of target genes for ETEC. YL and YQ participated in bacteria culture and DEC biochemical identification. QL participated in the design of the study. QH conceived of the study, and participated in its design and coordination and revised the manuscript. All authors read and approved the final manuscript.

## Additional file

## Supplementary Material

Additional file 1: Table S1. Reference and clinical strains used for multiplex real-time PCR assay. Click here for file
