# Supplementary material for: Rapid genetic typing of diarrheagenic Escherichia coli using a two-tube modified molecular beacon based multiplex real-time PCR assay and its clinical application
Source: Ann Clin Microbiol Antimicrob. 2014 Jul 15;13:30. doi: 10.1186/s12941-014-0030-8 (PMC4115161; doi:10.1186/s12941-014-0030-8)
Supplement: Additional file 1: Table S1. — Reference and clinical strains used for multiplex real-time PCR assay. [file s12941-014-0030-8-S1.docx]

**Supplementary Table 1. Reference and clinical strains used for multiplex real-time PCR assay**

| Reference strains/  Clinical strains | Number | Source | Virulence gene | | | | | | | |
| --- | --- | --- | --- | --- | --- | --- | --- | --- | --- | --- |
|  |  |  | Stp | Sth | Lt | aggR | eaeA | escV | stx1/stx2 | ipaH |
| *Acinetobacterbaumannii*  *Bacillus cereus*  *Bacillus subtilis*  *Campylobacter jejuni*  *Citrobacterfreudii*  *Enterobacteraerogenes*  *Enterobacter cloacae*  *Enterococcus faecalis*  *Enterobactersakazakii*  *Escherichia coli*  *E. coli O157:H7*  *Garcinia micrococcus*  *Klebsiellapneumoniae*  *Listeria monocytogenes*  *Proteus mirabilis*  *Proteus vulgaris*  *Pseudomonas aeruginosa*  *Serratiamarcescens*  *Shigellaboydii*  *Shigelladysenteriae*  *Shigellaflexneri*  *Shigellasonnei*  *Staphylococcus aureus* | 1  1  1  1  1  1  1  1  1  1  1  1  1  1  1  1  1  1  1  1  1  1  1 | ATCC 19606  ATCC 11778  ATCC 6633  ATCC 33292  CMCC 48001  CMCC 45103  CMCC 45301  ATCC 29212  ATCC 51329  ATCC 25922  ATCC 35150  ATCC 9341  ATCC 700603  ATCC 19114  ATCC 29906  CMCC 49001  ATCC 27853  CMCC 41002  CMCC 51265  CMCC 51376  ATCC12022  CMCC 51081  ATCC 27664 | -  -  -  -  -  -  -  -  -  -  -  -  -  -  -  -  -  -  -  -  -  -  - | -  -  -  -  -  -  -  -  -  -  -  -  -  -  -  -  -  -  -  -  -  -  - | -  -  -  -  -  -  -  -  -  -  -  -  -  -  -  -  -  -  -  -  -  -  - | -  -  -  -  -  -  -  -  -  -  -  -  -  -  -  -  -  -  -  -  -  -  - | -  -  -  -  -  -  -  -  -  -  +  -  -  -  -  -  -  -  -  -  -  -  - | -  -  -  -  -  -  -  -  -  -  +  -  -  -  -  -  -  -  -  -  -  -  - | -  -  -  -  -  -  -  -  -  -  +  -  -  -  -  -  -  -  -  -  -  -  - | -  -  -  -  -  -  -  -  -  -  -  -  -  -  -  -  -  -  +  +  +  +  -- |

| Reference strains/  Clinical strains | Number | Source | Virulence gene | | | | | | | |
| --- | --- | --- | --- | --- | --- | --- | --- | --- | --- | --- |
|  |  |  | Stp | Sth | Lt | aggR | eaeA | escV | stx1/stx2 | ipaH |
| *Streptococcus hemolytic-β*  *Salmonella aberdeen*  *Salmonella abortus-equi*  *Salmonella adelaide*  *Salmonella anatum*  *Salmonella cholerae-suis*  *Salmonella coli*  *Salmonella dublin*  *Salmonella enteritidis*  *Salmonella gallinarum*  *Salmonella infantis*  *Salmonella javiana*  *Salmonella kentucky*  *Salmonella litchfield*  *Salmonella london*  *Salmonella Manchester*  *Salmonella manhattan*  *Salmonella meleagridis*  *Salmonella minnesota*  *Salmonella moscow*  *Salmonella muenchen*  *Salmonella newport*  *Salmonella oranienburg*  *Salmonella paratyphi A* | 1  1  1  1  1  1  1  1  1  1  1  1  1  1  1  1  1  1  1  1  1  1  1  1 | CMCC 32210  CMCC 50313  CMCC 47717  CMCC 50065  CMCC 50083  CMCC 47649  CMCC 50809  CMCC 50042  CMCC 50040  CMCC 50770  CMCC 50341  CMCC 50364  CMCC 50794  CMCC 50810  CMCC 50310  CMCC 50380  CMCC 50151  CMCC 50329  CMCC 50061  CMCC 50044  CMCC 50125  CMCC 50029  CMCC 50379  CMCC 50001 | **-**  **-**  **-**  **-**  **-**  **-**  **-**  **-**  **-**  **-**  **-**  **-**  **-**  **-**  **-**  **-**  **-**  **-**  **-**  **-**  **-**  **-**  **-**  **-** | **-**  **-**  **-**  **-**  **-**  **-**  **-**  **-**  **-**  **-**  **-**  **-**  **-**  **-**  **-**  **-**  **-**  **-**  **-**  **-**  **-**  **-**  **-**  **-** | **-**  **-**  **-**  **-**  **-**  **-**  **-**  **-**  **-**  **-**  **-**  **-**  **-**  **-**  **-**  **-**  **-**  **-**  **-**  **-**  **-**  **-**  **-**  **-** | **-**  **-**  **-**  **-**  **-**  **-**  **-**  **-**  **-**  **-**  **-**  **-**  **-**  **-**  **-**  **-**  **-**  **-**  **-**  **-**  **-**  **-**  **-**  **-** | **-**  **-**  **-**  **-**  **-**  **-**  **-**  **-**  **-**  **-**  **-**  **-**  **-**  **-**  **-**  **-**  **-**  **-**  **-**  **-**  **-**  **-**  **-**  **-** | **-**  **-**  **-**  **-**  **-**  **-**  **-**  **-**  **-**  **-**  **-**  **-**  **-**  **-**  **-**  **-**  **-**  **-**  **-**  **-**  **-**  **-**  **-**  **-** | **-**  **-**  **-**  **-**  **-**  **-**  **-**  **-**  **-**  **-**  **-**  **-**  **-**  **-**  **-**  **-**  **-**  **-**  **-**  **-**  **-**  **-**  **-**  **-** | **-**  **-**  **-**  **-**  **-**  **-**  **-**  **-**  **-**  **-**  **-**  **-**  **-**  **-**  **-**  **-**  **-**  **-**  **-**  **-**  **-**  **-**  **-**  **-** |

| Reference strains/  Clinical strains | Number | Source | Virulence gene | | | | | | | |
| --- | --- | --- | --- | --- | --- | --- | --- | --- | --- | --- |
|  |  |  | Stp | Sth | Lt | aggR | eaeA | escV | stx1/stx2 | ipaH |
| *Salmonella paratyphi B*  *Salmonella paratyphi C*  *Salmonella typhi*  *Salmonella typhi-suis*  *Salmonella typhimurium*  *Vibrio cholerae*  *Vibrio parahaemolyticus*  *Vibrio vulnificus*  *Yersinia enterocolitica*  *Enterotoxigenic E.coli*  *Enteroaggregative E.coli*  *Enteroinvasive E.coli*  *Enterohemolysin E.coli*  *Enteropathogenic E.coli*  *Enterotoxigenic E.coli*  *Enteroaggregative E.coli*  *Enteroinvasive E.coli*  *Enterohemolysin E.coli*  *Enteropathogenic E.coli* | 1  1  1  1  1  1  1  1  1  1  1  1  1  1  16  8  8  8  10 | CMCC 50004  CMCC 50017  CMCC 50097  CMCC 50734  CMCC 50013  ATCC 14101  ATCC17082  ATCC 27562-2  CMCC 52201  ATCC 35401  ATCC 33780  ATCC 43893  ATCC 35150  ATCC 43887  Shenzhen CDC Shenzhen CDC Shenzhen CDC Shenzhen CDC Shenzhen CDC | **-**  **-**  **-**  **-**  **-**  **-**  **-**  **-**  **-**  **+**  **-**  **-**  **-**  **-**  **4**  **-**  **-**  **-**  **-** | **-**  **-**  **-**  **-**  **-**  **-**  **-**  **-**  **-**  **+**  **-**  **-**  **-**  **-**  **6**  **-**  **-**  **-**  **-** | **-**  **-**  **-**  **-**  **-**  **-**  **-**  **-**  **-**  **+**  **-**  **-**  **-**  **-**  **6**  **-**  **-**  **-**  **-** | **-**  **-**  **-**  **-**  **-**  **-**  **-**  **-**  **-**  **-**  **+**  **-**  **-**  **-**  **-**  **8**  **-**  **8**  **10** | **-**  **-**  **-**  **-**  **-**  **-**  **-**  **-**  **-**  **-**  **-**  **-**  **+**  **+**  **-**  **-**  **-**  **8**  **10** | **-**  **-**  **-**  **-**  **-**  **-**  **-**  **-**  **-**  **-**  **-**  **-**  **+**  **+**  **-**  **-**  **-**  **8**  **-** | **-**  **-**  **-**  **-**  **-**  **-**  **-**  **-**  **-**  **-**  **-**  **-**  **+**  **-**  **-**  **-**  **-**  **-**  **-** | **-**  **-**  **-**  **-**  **-**  **-**  **-**  **-**  **-**  **-**  **-**  **+**  **-**  **-**  **-**  **-**  **8**  **-**  - |

**Supplementary** Table2. Primer sequences used for conventional PCR

| Target genes | Primer sequence | Sequence(5’-3’) | Conc^a^(μM) | product size (bp) |
| --- | --- | --- | --- | --- |
| *st*  *lt*  *aggR*  *ipaH*  *eaeA*  *stx* | *st*-F  *st*-R  *lt*-F  *lt*-R  *aggR*-F  *aggR*-R  *ipaH*-F  *ipaH*-R  *eaeA*-F  *eaeA*-R  VT-com-F  VT-com-R | TTAATAGCACCCGGTACAAGCAGG  CCTGACTCTTCAAAAGAGAAAATTAC  TCTCTATGTGCATACGGAGC  CCATACTGATTGCCGCAAT  GTATACACAAAAGAAGGAAGC  ACAGAATCGTCAGCATCAGC  GTTCCTTGACCGCCTTTCCGATACCGTC  GCCGGTCAGCCACCCTCTGAGAGTAC  CCCGAATTCGGCACAAGCATAAGC  CCCGGATCCGTCTCGCCAGTATTCG  GAGCGAAATAATTTATATGTG  TGATGATGGCAATTCAGTAT | 0.5  0.5  0.25  0.25  0.25  0.25  0.125  0.125  0.125  0.125  0.25  0.25 | 147  322  254  619  881  518 |
